# Primary Esophageal Lymphoma: A Histopathological Experience from Two Tertiary Hospitals, Western Saudi Arabia

**DOI:** 10.1155/2023/7302344

**Published:** 2023-01-30

**Authors:** Jaudah Al-Maghrabi, Sahar Al-Maghrabi

**Affiliations:** ^1^Department of Pathology, Faculty of Medicine, King Abdulaziz University, Jeddah, Saudi Arabia; ^2^Department of Pathology, King Faisal Specialist Hospital & Research Center, Jeddah, Saudi Arabia; ^3^Faculty of Medicine, King Abdulaziz University, Jeddah, Saudi Arabia

## Abstract

**Background:**

Primary esophageal lymphoma (PEL) is a rare disorder. The objective of this study was to document the clinicopathological features of PEL at two tertiary hospitals in the western region of the Kingdom of Saudi Arabia.

**Methods:**

All PELs diagnosed between May 2002 and June 2022 were retrieved. Histopathological and immunohistochemical slides were reviewed. Additional immunohistochemistry stains were performed in selected cases. Follow-up data were collected.

**Results:**

There were only eight cases of PEL in the records of the two hospitals. The age of the patients ranged between 50 and 74 years (median 62 years and mean 62.5 years). There were six males (80%) and two females (20%). None of the patients were immunocompromised or had human immunodeficiency virus (HIV) infection. The clinical manifestation included dysphagia and loss of weight. Six cases were diffuse large B-cell lymphoma (DLBCL), and two were low-grade mucosa-associated lymphoid tissue lymphoma.

**Conclusion:**

PEL is an extremely rare disease with male predominance. DLBCL is the most common pathological type in our community. There was no relation to immune status or HIV infection in this series. Clinical presentations were typically dysphagia with weight loss. Further reporting of PEL cases might help explain this disease and improve its diagnosis and management.

## 1. Introduction

Esophageal lymphoma is extremely rare and frequently misdiagnosed. It accounts for less than 1% of all gastrointestinal lymphomas. Esophageal involvement is often developed as a result of spread from the adjacent stomach or mediastinal lymph nodes. Primary esophageal lymphoma (PEL) is even rarer. Little is known about the pattern of PEL in Saudi Arabia, and data are limited to a few case reports [1, 2]. Therefore, additional information is required to better understand this entity. The objective of this study was to document the clinicopathological features of PEL at two tertiary hospitals in the western region of the Kingdom of Saudi Arabia (KSA).

## 2. Methods

The study was carried out at King Abdulaziz University Hospital (KAUH) and King Faisal Specialist Hospital & Research Centre (KFSH&RC), Jeddah, Saudi Arabia. These are the two main referral hospitals in western Saudi Arabia. The study included all cases diagnosed as PELs. The definition proposed by Krol et al. [3] was used to define primary extranodal non-Hodgkin lymphoma (NHL), which is a liberal definition of primary extranodal NHL that includes all patients who present with NHL that apparently originated at an extranodal site, even in the presence of disseminated disease, as long as the extranodal component is clinically dominant. Therefore, this series included all patients who present with NHL originating at the esophagus when it is clinically dominant. The collected clinical data include age at presentation, gender, clinical features, treatment, and outcome. The immunohistochemistry slides were reviewed, and more immunohistochemistry markers were performed in selected cases. The minimum immunohistochemistry panel included CD45, CD20, CD3, BCL-2, BCL-6, CD10, MUM-1, and KI-67. Additional panels were added in selected cases and included vimentin, pankeratin, desmin, CD117, DOG-1, and CD34. Histopathological classification of lymphomas was according to the 2017 World Health Organization criteria [4]. DLBCLs were subclassified according to the Hans algorithm to germinal center B-cells (GCB) and non-GCB (non-GCB) that depend on the pattern of immunohistochemical expression for CD10, MUM-1, and BCL-6 [5]. The project covered cases diagnosed between May 2002 and June 2022. The study was approved by the Research Committee of the Biomedical Ethics Unit at our institution (Reference No. 34-22). Informed signed consent was waived by the Biomedical Ethics Committee as the research project used archived material. The practices followed were consistent with the 1975 Helsinki Declaration, as amended in 2013. A review of morphology and additional immunohistochemistry markers allowed for the re-classification of older cases into currently accepted diagnostic categories.

## 3. Results

**Figure 1:**
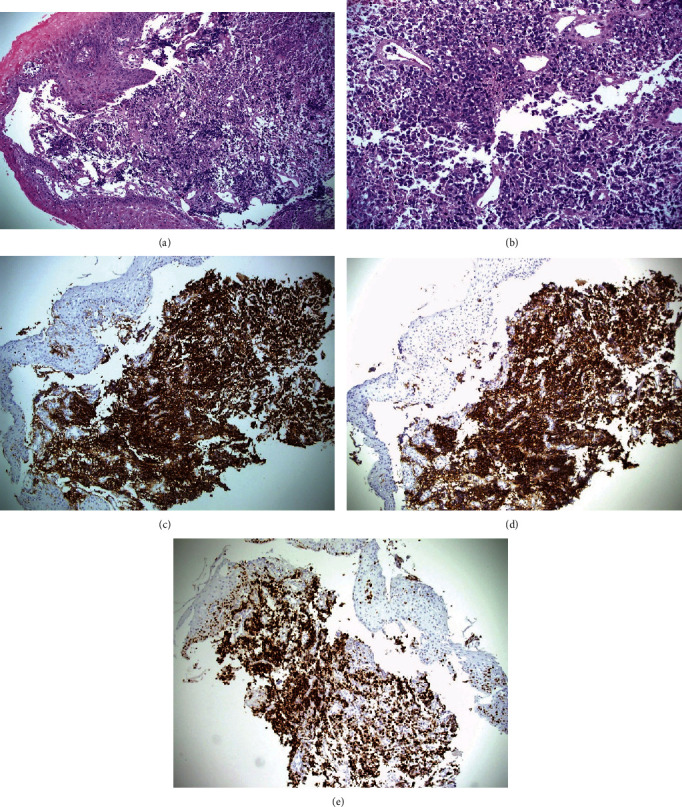
(a) Section of esophageal mass showing submucosal infiltration of lymphoma cells (hematoxylin and eosin, 200×). (b) Higher power of the same case; reveal large lymphoma cells surrounding some blood vessels (hematoxylin and eosin, 400×). (c) Diffuse large B-cell lymphoma expressing CD45 (immunohistochemistry stain, 200×). (d) Diffuse large B-cell lymphoma expressing CD20 (immunohistochemistry stain, 200×). (e) Diffuse large B-cell lymphoma with positive nuclear staining in 80% of tumor cells for KI-67 (immunohistochemistry stain, 200×).

There were only eight identified cases of PEL diagnosed at both institutions and their clinicopathological features are summarized in Table 1. One of the cases had been published previously [1]. Six cases were diagnosed based on endoscopic biopsies and two after resection. The age of the patients ranged between 50 and 74 years (median 62 years and mean 62.5 years). There were six males (80%) and two females (20%). The clinical manifestation included dysphagia (eight patients), loss of weight (five patients), and epigastric pain (one patient), and heartburn (one patient). Seven patients had an exophytic mass, and one had an ulcer. Seven patients underwent endoscopic biopsies. The first biopsies were diagnostic in five of them, and repeated biopsies were required to make the diagnosis in one case. In one case the biopsy was inconclusive. Therefore, in two cases the diagnosis was made after surgical resection. Six cases were diffuse large B-cell lymphoma (DLBCL), and two low-grade mucosa-associated lymphoid tissue (MALT) lymphoma. DLBCLs show diffuse infiltration of large cells with no low-grade small cell component to suggest transformation (Figures 1(a), 1(b), 1(c), 1(d), and 1(e)). One case was originally labeled as non-Hodgkin's B-cell lymphoma, medium to large sized cells and reclassified as DLBCL. They showed positive staining for CD45 and CD20, and negative for CD3. Ki-67 varied between 50% and 85% of tumor cells. MALT lymphoma is composed of submucosal proliferation of small centrocyte-like cells. They showed positive staining for CD45, CD20, and bcl-2, and negative for CD3, CD10, CD5, CD23, BCL-6, and cyclin-D1. Five of DLBCLs turned out of non-GCB. No further material was available from the sixth one to perform immunohistochemistry studies for subtyping. None of the patients in this study were immunocompromised or acquired immunodeficiency syndrome (AIDS) patients. Three patients with DLBCLs were treated with rituximab, cyclophosphamide, doxorubicin, vincristine, and prednisone (R-CHOP), one patient treated with rituximab, cyclophosphamide, vincristine sulfate, and prednisone (R-CVP), one patient deteriorated and died before the initiation of chemotherapy, and one transferred to another institution and no details available on management. The patients with MALT lymphoma were treated with surgical resection.

Radiological evaluation by computed tomography (CT) showed thickening of the esophageal wall with mass lesion causing luminal narrowing in seven patients. The patient with the ulcer also had esophageal wall thickening. The results of a barium esophagogram were available for three patients and showed localized, but significant narrowing of the esophagus.

## 4. Discussion

PEL is an extremely rare disease, and information is limited to a few case reports and small case series. There are only two PEL previously reported from the KSA [1, 2]. In the current study, new seven additional cases of PEL were reported, and all were NHL. The age of the patients in the current study ranged between 50 and 74 years. There were six males and two females. Six cases were DLBCL, and two were low-grade MALT lymphoma. Quiroga-Centeno et al. [6] showed that male gender predominates via data obtained from Surveillance, Epidemiology, and End Results (SEER) study. The mean age at presentation of MALT lymphoma patients was 78.5 years, and the mean age at presentation of patients of the other histological type was 65 years. These data demonstrated that DLBCL is the most common pathological type followed by lymphoma of MALT type. However, in the systematic review published in the same study, MALT-type lymphoma was the most frequent among cases reported in the literature. They suggested that this difference between SEER data and data from the literature review regarding the most common histopathological type of PEL is probably explained by geographical differences because most of the PEL case reports were in Asian patients (47.8%) where MALT may predominate.

While our current study is small, six of the eight patients demonstrated DLBCL type, which suggested that esophageal DLBCL is more common than MALT lymphoma in KSA. There is no consensus standard therapy for PEL; however, most patient in the systemic review received chemotherapy using cyclophosphamide, doxorubicin, vincristine, and prednisone or R-CHOP. Some had surgical resection or combination approach. A combination of chemotherapy and radiotherapy seems to be the preferred approach of treatment. Surgical and endoscopic resections could be a reasonable alternative approach according to histological type and initial site of the tumor. The median overall survival was 12 months with excellent prognosis of MALT lymphoma compared with DLBCL [6]. Other histological types of PEL have also been described in the literature including Burkitt's lymphoma [7], mantle cell [8], anaplastic [9, 10], T-cell lymphoma [11], Hodgkin's lymphoma [2, 12], and true histiocytic lymphoma [13].

In the previously reported Hodgkin's lymphoma case from KSA [1, 2], the patient was treated with chemotherapy. The follow-up showed no evidence of recurrence through the date of publication.

PEL may pose a diagnostic dilemma because the clinical manifestation as well as radiological and endoscopic findings of PEL are variable and nonspecific. This makes the diagnosis challenging. It is important to be differentiated from benign soft tissue tumor, such as leiomyoma or gastrointestinal stromal tumor and other non-hemopoietic malignant tumors, such as carcinomas because of different management.

The pathogenesis of PEL is unknown, and the role of viruses including human immunodeficiency virus (HIV), Epstein-Barr virus, and hepatitis C is controversial. HIV infection is the more probable risk factor [14, 15]. PEL is more commonly seen in immunosuppressive conditions (such as AIDS), medications, and transplantation [15, 16]. In the literature, AIDS-related PEL should be considered in the differential diagnosis of AIDS patients presenting with dysphagia and esophageal mass. Inayat et al. reported HIV infection in 5 out of 15 PEL cases [14]. None of the patients in this series were immunocompromised or had HIV infection. In the Hodgkin' lymphoma case reported before from KSA, HIV testing was not reported [2]. This probably indicates that HIV is unlikely to play a significant role in the pathogenesis of PEL in our community. There are some reports of MALT lymphoma of lower esophagus associated with *Helicobacter pylori* infection that even responded to *H. pylori* eradication therapy [17]. However, most of the reported MALT lymphomas in the esophagus were negative for *H. pylori* regardless of the tumor locations in the esophagus [18, 19]. Furthermore, rapid urase tests for *H. pylori* were negative in six patients in this series and was not done in two cases. No *H. pylori* could be identified in any of the biopsies in this series. This indicates that the etiology of PEL needs to be explored further.

The prognosis of PEL depends significantly on the histopathological subtypes. The response of PEL of DLBCL type to R-CHOP with or without radiotherapy is relatively good and achieves complete remission in most cases [14]. In this study, the three patients who received R-CHOP survived for 25, 22, and 15 months. The patient who received R-CVP responded initially to chemotherapy and developed large tracheoesophageal fistula, pneumonia, and died within 5 months of diagnosis. One patient with DLBC did not receive any chemotherapy because of the rapid clinical progression and passed away. The sixth DLBCL patient transferred to another hospital and no available data on the management details. The two patients with MALT lymphoma were treated with surgical resection. Surgical treatment, radiation therapy, or chemotherapy were different treatment strategies in the treatment of PEL of MALT type. However, the optimal treatment approach is unknown due to the rarity of the disease and slow disease progression.

The patient with MALT survived for 17 and 38 months and was then lost to follow-up. The prognosis of patients with PEL of low-grade MALT is generally much better than other histological types. The 1- and 2-year overall survival rates were 62.5% and 25%, respectively, during follow-up in the current study. Patients with PEL may present with tracheoesophageal fistula or develop this kind of fistula as a complication as what happened in our case [20, 21].

The radiological features of PEL in the literature are variable and non-specific. In this study, seven patients had an exophytic mass and one had an ulcer. Different radiological features have been described in PEL including polypoid masses with or without ulceration, submucosal masses, nodules, diffuse intramural submucosal tumor, ulcerated narrowing, varicoid appearance, aneurysmal dilatation, and achalasia-like patterns with a narrowed distal esophagus [12, 14].

The rarity of PEL and the non-specific radiological features make the pathological diagnosis a challenging process especially in small biopsies. The pathological differential diagnosis depends on the lymphoma histological subtypes. Immunohistochemistry and molecular study for clonality confirmation will help confirm the diagnosis [22]. In the cases shown here, the diagnosis was made based on the morphology and characteristic immunohistochemical profile. The pathological diagnosis in most of the cases of PEL was made through endoscopic biopsy with some cases diagnosed only after surgical resection of the tumor [1, 10]. Diagnosis of PEL can be also made by cytologic evaluation using endoscopic ultrasound-guided fine needle aspiration (FNA) [23, 24]. However, no FNA cytology was performed in the cases of this series.

## 5. Conclusion

PEL is a rare disease with male predominance. DLBCL is the most common pathological type in our community. It looks that there is no relation of PEL to immune status or HIV infection in community. Clinical presentations were typically dysphagia with weight loss. Clinical and radiologic findings are not specific, and diagnosis is usually made on endoscopic biopsies. Further reporting of PEL cases might help explain this disease and improve its diagnosis and management.

## Figures and Tables

**Table 1:** Summary of the PEL cases diagnosed at KAUH and KFSH&RC, Jeddah.

	Age/sex	Site	Clinical presentation	Diagnosis	BM	Tumor gross	Follow-up (months)	Treatment/outcome
1	68/M	Lower esophagus	Dysphagia and weight loss	DLBCL	−VE	Polypoid mass	15	R-CHOP
2	56/M	Lower esophagus	Dysphagia	MALT	−VE	Exophytic mass	38	Surgical resection
3	62/M	Lower esophagus	Dysphagia and weight loss	DLBCL	NA	Exophytic mass	1	Patient deteriorated, developed pneumonia, and died before initiation of chemotherapy.
4	74/F	Upper esophagus	Dysphagia and weight loss	DLBCL	−VE	Exophytic mass	5	R-CVP: She initially responded to chemotherapy. Developed large tracheoesophageal fistula, pneumonia, and died
5	58/M	Lower esophagus	Dysphagia, weight loss, and epigastric pain	DLBCL	−VE	Ulcer	25	R-CHOP
6	65/M	Lower esophagus	Dysphagia	DLBCL	NA	Exophytic mass	22	R-CHOP
7	63/F	Lower esophagus	Dysphagia and weight loss	DLBCL	−VE	Exophytic mass	1	NA
8	50/M	Lower esophagus	Dysphagia and heartburn	MALT	NA	Exophytic mass	17	Surgical resection

−VE: negative; DLBCL: diffuse large b-cell lymphoma; MALT: mucosa-associated lymphoid tissue; BM: bone marrow; NA: not available.

## Data Availability

Data supporting this research article are available from the corresponding author or first author on reasonable request.
